# The Effectiveness of In-Hospital Continuous Masking Policies In Reducing Respiratory Viral Infection Risk: A Systematic Review

**DOI:** 10.1017/ash.2025.401

**Published:** 2025-09-24

**Authors:** Qandeel Shafqat, Elissa Rennert-May, Stephanie Smith, Devika Dixit, Connor Rutherford, Jessalyn Almond, Oscar Larios, John Conly, Jenine Leal

**Affiliations:** 1 University of Calgary; 2 Alberta Health Services; 3 Foothills Medical Centre; 4 Alberta Health Services/University of Calgary

## Abstract

Respiratory viral infection (RVI) outbreaks pose a significant threat to health. They are associated with patient morbidity and mortality, staff absenteeism, and financial burden on the healthcare system. There is a need for strategies to reduce RVI transmission in hospitals. One proposal is implementation of continuous masking policies. However, the effectiveness of such policies in mitigating RVI spread is unclear. We conducted a systematic review of the literature to determine the effectiveness of continuous masking in reducing the incidence and transmission of RVIs amongst patients and healthcare workers (HCWs) in hospitals. We systematically searched for original articles published between 2000-2024 according to a pre-determined search criterion. Studies were screened by two reviewers in Covidence. One reviewer extracted the data from eligible studies into a pre-determined data extraction form. For studies that reported only count data, results were summarized narratively. Meta-analysis to pool unadjusted or adjusted outcome measures for studies that report a statistical comparison between masking policies and transmission of infections will be considered if appropriate. Joanna Briggs Institute tools will be used for critical appraisal. 3691 studies were identified. 17 met eligibility criteria. 12 studies were conducted in single-center adult hospitals. 4 studies were conducted in pediatric hospitals, with 2 in neonatal centers. One study was conducted on a hospital system. The studied infections were influenza A/B, parainfluenza 1-3, adenovirus, respiratory syncytial virus (RSV), traditional human coronavirus strains, human metapneumovirus, SARS-CoV-2, and rhinovirus/enterovirus. Eight studies assessed the impact of a masking policy on infection rate in patients. All 8 reported masking policies reduce RVI transmission in patients. 9 studies assessed the impact of a masking policy on infection rate in HCWs. 7 were associated with reductions in RVI transmission in HCWs, whereas 2 showed no statistically significant change. The studies identified in this systematic review were associated with a reduction in RVI transmission with the use of continuous masking amongst patients. The evidence for continuous masking was less consistent for preventing RVI transmission amongst HCW with two studies reporting it was not effective. Our findings suggest that masking policies may play a role in RVI prevention but there are significant limitations with the use of observational design and masking in conjunction with other prevention measures. However, assessment of the quality of the papers is pending. Future directions will include assessing secondary outcomes like masking adherence and assessing adjusted analyses form confounding which are critically important.

